# A Robust ROS Generation and Ferroptotic Lipid Modulation Nanosystem for Mutual Reinforcement of Ferroptosis and Cancer Immunotherapy

**DOI:** 10.1002/adhm.202401502

**Published:** 2024-10-01

**Authors:** Chao Jiang, Wenxi Li, Jie Yan, Xinying Yu, Yuzhao Feng, Bei Li, Yuan Liu, Yunlu Dai

**Affiliations:** ^1^ Cancer Center and Institute of Translational Medicine Faculty of Health Sciences University of Macau Macau SAR 999078 China; ^2^ MoE Frontiers Science Center for Precision Oncology University of Macau Macau SAR 999078 China; ^3^ Zhejiang Cancer Hospital Hangzhou Institute of Medicine (HIM) Chinese Academy of Sciences Hangzhou Zhejiang 310022 China

**Keywords:** cancer immunotherapy, ferroptosis, ferroptotic lipids, immunogenic cell death, reactive oxygen species

## Abstract

Ferroptosis initiation is often utilized for synergistic immunotherapy. While, current immunotherapy is limited by an immunosuppressive tumor microenvironment (TME), and ferroptosis is limited by insufficient reactive oxygen species (ROS) and ferroptotic lipids in tumor cells. Here, an arachidonic acid (AA) loaded nanosystem (CTFAP) is developed to mutually reinforce ferroptosis and cancer immunotherapy by augmenting ROS generation and modulating ferroptotic lipids. CTFAP is composed of acid‐responsive core calcium peroxide (CaO_2_) nanoparticles, ferroptotic lipids sponsor AA, tetracarboxylic porphyrin (TCPP) and Fe^3+^ based metal‐organic framework structure, and biocompatible mPEG‐DSPE for improved stability. Once endocytosed by tumor cells, CTFAP can release oxygen (O_2_) and hydrogen peroxide (H_2_O_2_) in the acidic TME, facilitating TCPP‐based sonodynamic therapy and Fe^3+^‐mediated Fenton‐like reactions to generate substantial ROS for cell ferroptosis initiation. The immunogenic cell death (ICD) after ferroptosis promotes interferon γ (IFN‐γ) secretion to up‐regulate the expression of long‐chain family member 4 (ACSL4), cooperating with the released AA from CTFAP to accelerate the accumulation of lipid peroxidation (LPO) and thereby promoting ferroptosis in cancer cells.CTFAP with ultrasound treatment efficiently suppresses tumor growth, has great potential to challenges in cancer immunotherapy.

## Graphical abstract

To mutually promote ferroptosis and cancer immunotherapy, a CaO_2_ core‐based nanosystem (CTFAP) is developed to augment reactive oxygen species generation for ferroptosis initiation. Immunogenic cancer cell death promotes dendritic cell maturation and stimulates systemic antitumor immune responses to secrete IFN‐γ. The released IFN‐γ further up‐regulates long‐chain family member 4 expression, cooperating with the released arachidonic acid from the nanosystem in turn promoting cell ferroptosis.

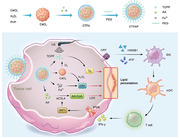

## 1 Introduction

Currently, despite significant strides in traditional cancer treatment, cancer is still one of the primary causes of death worldwide.^[^ ^1^ ^]^ Immunotherapy exhibited great potential in cancer treatment by eliciting a sustained immune response.^[^ ^2^ ^]^ Several cancer immunotherapeutic strategies such as immune vaccines, immune checkpoint blockers, and cytokine drugs, have succeeded in clinical trials.^[^ ^3^ ^]^ However, the effectiveness of cancer immunotherapy is hampered by the immunosuppressive tumor microenvironment (TME) and immune escape, which preclude its benefits to all patients.^[^ ^4^ ^]^ Several studies have demonstrated that ferroptosis is conducive to triggering immunogenic cell death (ICD) and releasing damage‐associated molecular patterns (DAMPs) to promote immunotherapy.^[^ ^5^ ^]^ These DAMPs can regulate the immune‐relevant inflammatory response and further stimulate dendritic cells (DCs) maturation and cytotoxic T lymphocyte cells (CTLs) activation.^[^ ^6^ ^]^ Interferon γ (IFN‐γ) released from activated CTLs promotes tumor cell ferroptosis by downregulating the expression of SLC7A11,^[^ ^7^ ^]^ a subunit of the cysteine/glutamate anti‐transporter system. Decreasing the expression of SLC7A11 results in reduced glutathione (GSH) production and glutathione peroxidase 4 (GPX4) expression, thereby promoting ferroptosis.^[^ ^8^ ^]^ Therefore, enhancing ferroptosis emerges as a promising strategy for synergistic immunotherapy.

**Scheme 1:**
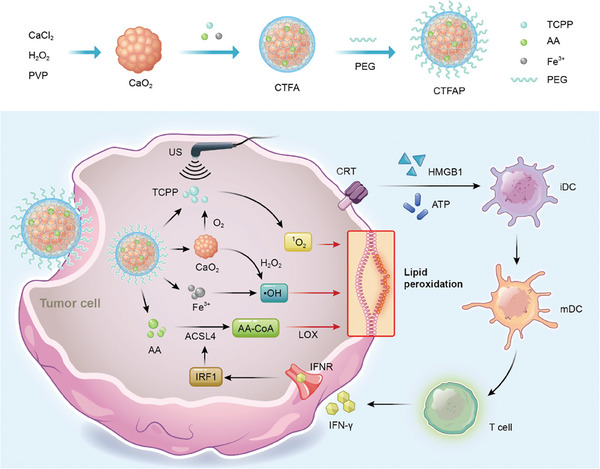
Illustration of the synthesis procedure and therapeutic mechanism of CTFAP nanoparticles. Upon being endocytosed by tumor cells, CTFAP releases both O_2_ and H_2_O_2_, which serve as substrates for TCPP‐based SDT to generate ^1^O_2_ and Fe^3+^‐mediated Fenton‐like reaction to produce •OH, respectively. The generated reactive oxygen species subsequently accelerates intracellular ferroptosis‐mediated ICD and triggers the release of IFN‐γ, which can further up‐regulate the expression of ACSL4. Meanwhile, the AA released from CTFAP can be converted into AA‐CoA for LPO accumulation with the catalysis of ACSL4 to promote ferroptosis. O_2_: oxygen, H_2_O_2_: hydrogen peroxide, TCPP: tetracarboxylic porphyrin, SDT: sonodynamic therapy, ^1^O_2_: singlet oxygen, •OH: hydroxyl radicals, ICD: immunogenic cell death, ACSL4: long‐chain acyl‐CoA synthetase 4, IFN‐γ: interferon γ, AA: arachidonic acid, AA‐CoA: arachidonoyl‐CoA, LPO: lipid peroxidation.

Ferroptosis, as an iron‐dependent programmed cell death, is distinct from apoptosis, necrosis, and autophagy.^[^ ^9^ ^]^ Lipid peroxidation (LPO) and reactive oxygen species (ROS) accumulation are regarded as two typical characteristic features of ferroptosis.^[^ ^10^ ^]^ Thus, the increase of LPO and ROS content is favorable for the progression of ferroptosis in tumor cells.^[^ ^11^ ^]^ Ferroptotic lipids, such as arachidonoyl‐CoA (AA‐COA), serve as precursor for LPO.^[^ ^12^ ^]^ However, the direct delivery of fatty acids for the enhancement of ferroptosis is hindered by insufficient long‐chain acyl‐CoA synthetase 4 (ACSL4) in tumor cells,^[^ ^13^ ^]^ ACSL4 plays a crucial role in modulating ferroptosis by converting fatty acids into ferroptotic lipids.^[^ ^14^ ^]^ Fortunately, recent works have revealed that in addition to inhibiting cystine transportation, IFN‐γ can also facilitate the esterification of arachidonic acid (AA) by upregulating the expression of ACSL4.^[^ ^15^ ^]^ Thus, a targeting AA metabolism‐based strategy through phospholipase A2 (PLA2) and lipoxygenase (LOX) delivery has been developed to enhance immunogenic ferroptosis.^[^ ^16^ ^]^ PLA2 facilitates the release of free AA from phospholipids, which is then converted into AA‐CoA through IFN‐γ‐mediated ACSL4 activation, and ultimately into peroxidized lipid under the catalysis of LOX. Based on the connection between AA and ferroptosis, we posit that both enhancing ROS generation and AA delivery may constitute an effective strategy for mutually reinforcing ferroptosis and cancer immunotherapy. Peroxide, such as calcium peroxid (CaO_2_), is widely used to supply hydrogen peroxide (H_2_O_2_) for the Fenton reaction, which generates equivalent ROS.^[17]^ Inaddition, sonodynamic therapy (SDT) can also initiate ferroptosis by ultrasound (US)‐mediated sonosensitizer activation for ROS generation.^[18]^ Based on these two strategies for ROS generation, we have developed a robust ROS generation nanosystem loaded with AA (denoted as CTFAP) for mutually reinforcing ferroptosis and cancer immunotherapy. AS shown in Scheme 1, pH‐sensitive CaO_2_ nanoparties we synthesized and used as the template for preparing AA‐loaded CaO_2_@TCPP‐Fe (CTFA) nanoparticles. PEG‐coated CTFA (CTFAP) nanoparticles could further improve their biocompatibility and stability during blood circulation. Meanwhile, CTFAP nanoparticles excellent pH‐responsive oxygen (O_2_) and H_2_O_2_ production capacity, promoting the generation of singlet oxygen (^1^O_2_) and hydroxyl radicals (·OH) through sonosensitizer (TCPP) based SDT and Fe^3+^ mediated Fenton‐like reaction, respectively. The abunant ROS subsequently accelerates intracellular ferropotsis and stimulates a potent anti‐tumor immune response, promoting the secretion of IFN‐γ from activated CTLs. In return, the released IFN‐γ promotes the conversion of the released AA into AA‐CoA by up‐regulating ACSL4 expression, thereby enhancing the LPO accumulation for cell ferroptosis. This study provides a novel approach to enhance cancer‐immunogenic ferroptosis by boosting ROS generation, AA delivery, and IFN‐γ promoted ACSL4 expression, which holds great promise for cancer treatment.

## 2 Results and Discussions

### 2.1 Preparation and Characterization of CTFAP

**Figure 1:**
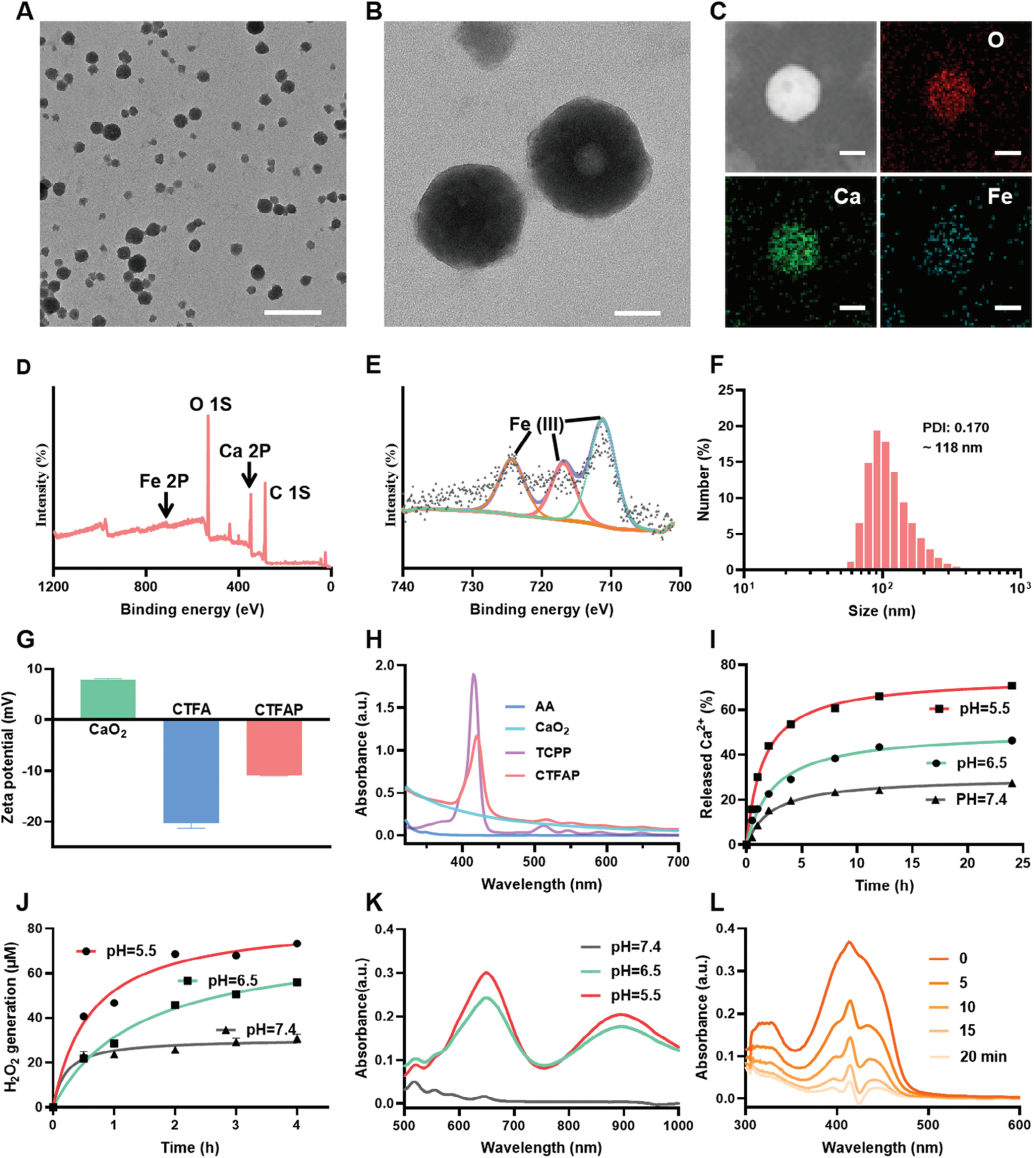
Physical and chemical characteristics of CTFAP. A) and B) TEM images of CTFAP. Scale bar: 500 and 50 nm, respectively. C) Element mapping of CTFAP. Scale bar: 50 nm. D) XPS spectrum of CTFAP. E) Fe 2p XPS high‐resolution spectrum. F) Hydrodynamic diameter distribution of CTFAP. G) Zeta potential of CaO_2_, CTFA, and CTFAP. H) UV‐vis spectra of AA, CaO_2_, TCPP, and CTFAP. I) Ca^2+^ and J) H_2_O_2_ release profiles of CTFAP in phosphate‐buffered saline with varying pH. K) UV‐vis spectra of TMB in CTFAP solution under different pH. L) UV‐vis absorption spectra of DPBF in CTFAP solution with various US irradiation durations.

The formation of CTFAP followed a three‐step approach (**Scheme** **1**). Initially, CaO_2_ nanoparticles were synthesized according to a wet‐chemical method with polyvinyl pyrrolidone (PVP) serving as a stabilizer.^[^ ^19^ ^]^ The successful preparation of CaO_2_ nanoparticles was confirmed through the reduction of permanganate (MnO_4_^−^) by peroxo groups.^[^ ^20^ ^]^ The purple color of the MnO_4_^−^ solution disappeared after the co‐incubation with CaO_2_ nanoparticles (Figure S1, Supporting Information). The size of CaO_2_ nanoparticles was ≈81 nm obtained from the transmission electron microscopy (TEM) image (Figure S2, Supporting Information). In the second step, the TCPP/Fe network was formed on the surface of CaO_2_ nanoparticles (CaO_2_@TCPP/Fe) by the strong coordination interaction between Fe^3+^ and carboxyl groups of TCPP. AA was introduced into the synthetic procedure to form CaO_2_@TCPP/Fe‐AA (CTFA). Finally, CTFA was modified by mPEG‐DSPE (CTFAP) to improve its stability and aqueous solubility; CTFAP exhibited good dispersion in different physiological media (Figure S3, Supporting Information). Additionally, the mPEG‐DSPE modified CaO_2_@TCPP/Fe (CTFP) was provided as the control group following the same synthesis method. The spherical CTFAP nanoparticles were observed from TEM images with a uniform particle size (**Figure** **1**A,B; Figure S4, Supporting Information). Energy dispersive X‐ray (EDX) spectroscopy elemental mapping and X‐ray photoelectron spectroscopy (XPS) confirmed the presence of O, Ca, and Fe elements in the composition of CTFAP (Figure 1C,D; Figure S5, Supporting Information). Fe 2p3/2 and Fe 2p1/2 binding energy peaks were found at 711.28 eV and 724.38 eV, respectively, with the satellite peak of Fe^3+^ identified at 716.88 eV, indicating the predominance of Fe3+ in CTFAP (Figure 1E). Dynamic light scattering (DLS) analysis recorded the hydrodynamic diameter of CTFAP nanoparticles was ≈118 nm (Figure 1F), consistent with the results in TEM results (Figure S4, Supporting Information). The surface modification of PEG caused a change in the zeta potentials of the nanoparticles from −20.3 to −10.9 mV (Figure 1G). A slight red shift could be observed both in the ultraviolet‐visible (UV‐vis) and fluorescence spectra of CTFAP (Figure 1H; Figure S6A, Supporting Information), and the absorption peak of carbonyl group shifted from 1700 to 1650 cm^−1^ in the Fourier transform infrared (FTIR) spectrum, suggesting the coordination reaction between TCPP and Fe^3+^ (Figure S6B, Supporting Information). Besides, the encapsulation efficiency of AA was quantified to be 34.18% by UV‐vis spectroscopy (Figure S7, Supporting Information).

### 2.2 In Vitro pH‐Responsive Decomposition and ROS Generation

Afterward, the pH‐responsive decomposition and ROS generation of CTFAP were meticulously examined. The release profiles of Ca^2+^ and Fe^3+^ from CTFAP in phosphate‐buffered saline (PBS) at various pH were measured using inductively coupled plasma mass spectrometry (ICP‐MS). After 24 h incubation in PBS pH 5.5, 70.7% of Ca^2+^ was released from CTFAP. In contrast, only 46.4% and 27.4% of Ca^2+^ were observed in PBS at pH 6.5 and 7.4, respectively (Figure 1I). Moreover, the amount of Fe^3+^ released from CTFAP in the buffer solution at pH 5.5 is about twice as much as at pH 7.4 (Figure S8, Supporting Information). Similarly, more H_2_O_2_ (73 µM) was detected at acid condition (pH 5.5), while less H_2_O_2_ was generated in PBS at pH 6.5 (56 µM) and 7.4 (30 µM) after 2 h incubation, respectively (Figure 1J). More visible results could be obtained from the TEM images; the CTFAP gradually degraded at acidic conditions (pH 6.5), while little change of the spherical nanoparticles could be observed in pH 7.4 PBS (Figure S9, Supporting Information). The elevated H_2_O_2_ could be converted into a higher toxic •OH in the presence of Fe^3+^ based on its Fenton‐like reaction. The generation of •OH was verified by using 3,3′5,5′‐tetramethylbenzidine (TMB), which can be oxidated into TMBox by •OH, resulting in an obvious change in color and characteristic absorption. As shown in Figure 1K, a characteristic absorption of TMBox could be found at acid conditions (pH = 5.5 and 6.5), indicating an increase in •OH generation due to the elevated H_2_O_2_ level at acidic conditions. Both CTFP and CTFAP could result in the generation of •OH at acid condition (pH 6.5) due to the existence of Fe^3+^, while negligible •OH was detected in the same buffer containing CaO_2_ and TMB or TMB only (Figure S10A, Supporting Information). There was a dose‐dependent for pH‐triggered •OH generation in the presence of CTFAP (Figure S10B, Supporting Information). In addition to H_2_O_2_, the hydrolysis of CaO_2_ can also release O_2_. Thus, the O_2_ release from CTFAP was recorded by oxygen meter in PBS (pH 6.5) (Figure S11, Supporting Information). Continuous production of O_2_ was observed in the solution containing CTFAP, which is favorable for O_2_ consumption based SDT. 1,3‐diphenylisobenzofuran (DPBF) was introduced to indicate the ^1^O_2_ generation from CTFAP exposed to US irradiation. The absorption of DPBF at 416 nm decreased dramatically during its oxidation by ^1^O_2_. The UV‐vis absorption spectra of the mixture of CTFAP and DPBF at different time points were monitored under US irradiation. With the increase of exposure time, the characteristic absorption of DPBF decreased, suggesting the generation of ^1^O_2_ (Figure 1L). These findings demonstrated that CTFAP could effectively enhance ROS production through a sufficient self‐supply of H_2_O_2_/O_2_, which is favorable to ferroptosis initiation.

### 2.3 In Vitro Anti‐Tumor Properties of CTFAP

**Figure 2:**
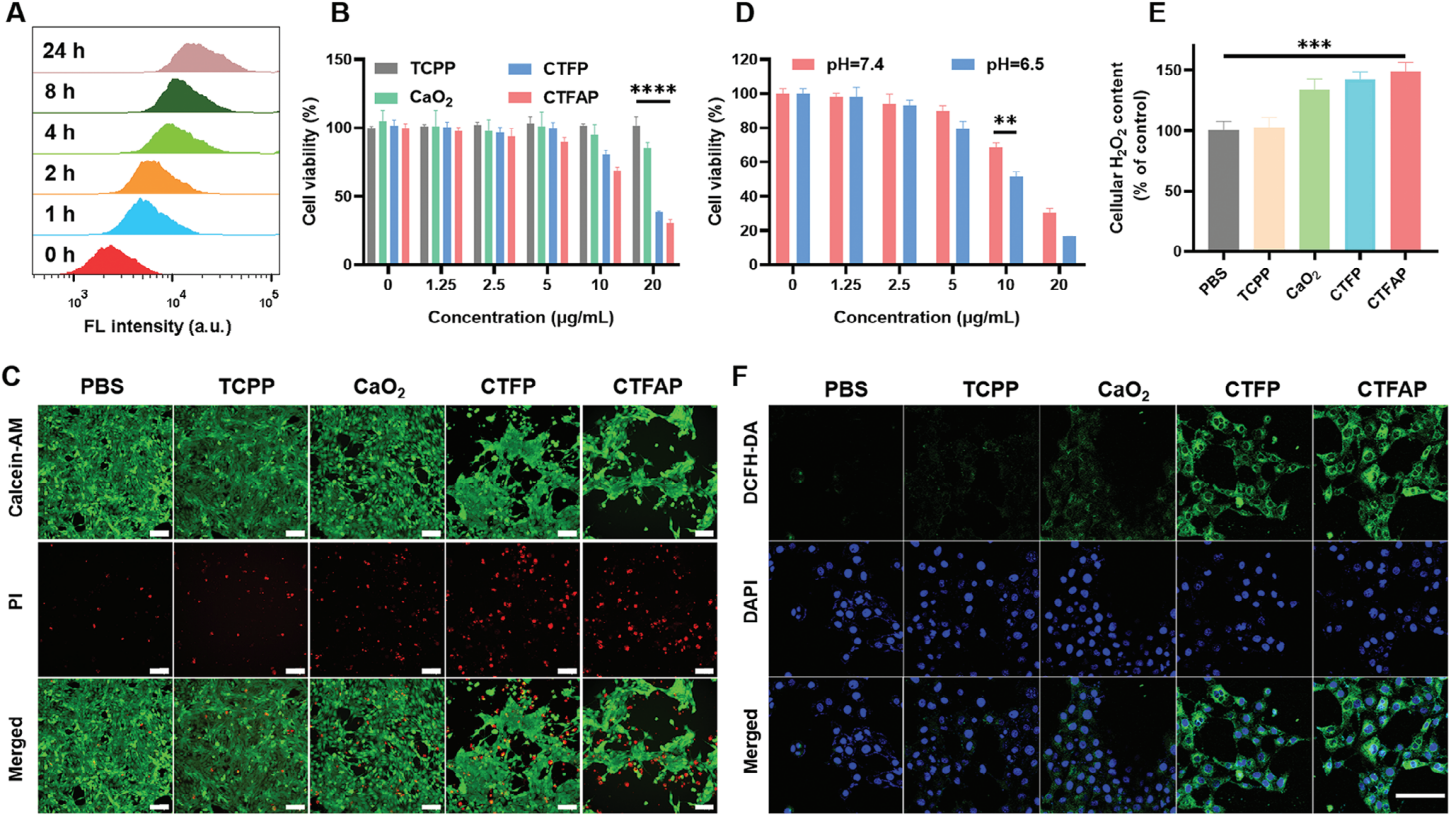
In vitro cytotoxicity of CTFAP. A) Flow cytometry analysis of 4T1 cells treated with CTFAP at varying times. B) Viabilities of 4T1 cells with various amounts of TCPP, CaO_2_, CTFP, and CTFAP. C) Microscopy images of live and dead cells stained by calcian‐AM (CA, green fluorescence) and propidium iodide (PI, red fluorescence). Scale bar: 100 µm. D) Viabilities of 4T1 cells incubated with varying concentrations of CTFAP under different pH (7.4 and 6.5). E) Intracellular H_2_O_2_ content in 4T1 cells with varying treatments. F) Microscopy images of DCFH‐DA stained 4T1 cells treated with PBS, TCPP, CaO_2_, CTFP, and CTFAP. Scale bar: 100 µm. (mean ± SD, n = 3), *p < 0.05, **p < 0.01, ***p < 0.001, and ****p < 0.0001.

Inspired by the outstanding capacity for ROS production, the cell‐killing ability of CTFAP was further studied on the 4T1 cell line. Initially, the cell uptake over time was detected by flow cytometry. CTFAP was effectively endocytosed by 4T1 cells and reached a platform after 8 h (**Figure** **2**A; Figure S12, Supporting Information). The cell cytotoxicity of CTFAP was subsequently measured using a CCK‐8 assay. The incubation of AA, TCPP, or CaO_2_ for 24 h resulted in negligible toxicity to 4T1 cells (Figure 2B; Figure S13, Supporting Information). However, the cell cytotoxicity of CTFP significantly increased due to the Fe^3+^‐mediated ferroptosis. The presence of AA further enhanced the killing ability of CTFAP, which may be attributed to the oxidization of AA by ROS to promote cell ferroptosis.^[^ ^15^ ^]^ Live/death staining results further confirmed the cell‐killing ability of CTFAP (Figure 2C). Significant red fluorescence and slight green fluorescence were observed in CTFP and CTFAP treated cells, indicating a higher number of dead cells in CTFP and CTFAP‐treated groups. Moreover, the normal human mammary epithelial (MCF‐10A) cells showed a high survival rates with the high doses of CTFAP, demonstrating the excellent biocompatibility of CTFAP (Figure S14, Supporting Information). Interestingly, less than 20% of cells survived in the CTFAP‐treated group at pH 6.5 (Figure 2D), which may contribute to the accelerated degradation of CTFAP under acidic conditions, resulting in elevated H_2_O_2_ levels and enhanced ROS generation. The capacity of intracellular H_2_O_2_ generation of CTFAP was further verified using an H_2_O_2_ kit. Intracellular H_2_O_2_ content in 4T1 cells increased by ≈50% in both CaO_2_, CTFP, and CTFAP‐treated groups (Figure 2E). The elevated levels of H_2_O_2_ may promote the ROS generation by Fe^3+^‐mediated Fenton‐like reaction. Subsequently, the intracellular ROS generation was measured by ROS indicator 2′,7′‐dichlorodihydrofluorescein diacetate (DCFH‐DA), nonfluorescent DCFH‐DA could be oxidized into 2′, 7′‐dichlorofluorescein (DCF) with green fluorescence. Slight green fluorescence was observed in TCPP and CaO_2_ treated groups, while both CTFP and CTFAP treated cells displayed varying intensities of green fluorescence due to the elevated H_2_O_2_ (Figure 2F). The most significant green fluorescence was observed in CTFP and CTFAP because of the Fe^3+^ mediated Fenton‐like reaction, confirming their excellent ability in ROS generation.

### 2.4 Investigation of CTFAP‐Induced Cell Ferroptosis

**Figure 3:**
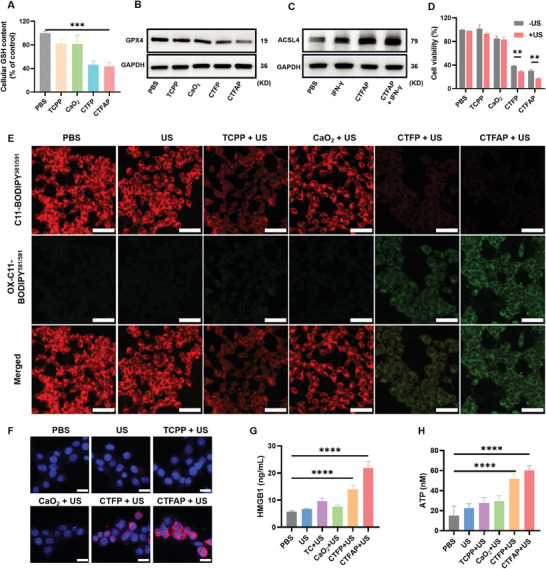
The ferroptosis initiation induced by CTFAP. A) Intracellular GSH content in treated 4T1 cells. Western blotting results of B) GPX4 and C) ACSL4 expression after various treatments. D) Viability of 4T1 cells treated by TCPP, CaO_2_, CTFP, and CTFAP with or without US irradiation. E) Microscopy images of C11‐BODIPY^581/591^ stained 4T1 cells. Scale bar: 50 µm. F) Microscopy images of cell membrane CRT exposure in 4T1 cells with various treatments. Scale bar: 20 µm. G) The detection of HMGB1 in cell culture supernatant. H) Release of ATP from treated 4T1 cells. (mean ± SD, n = 3), *p < 0.05, **p < 0.01, ***p < 0.001, and ****p < 0.0001.

Ferroptosis is a regulated form of cell death.^[^ ^9d^ ^]^ To detect whether ferroptosis occurred, the typical hallmarks of ferroptosis including ROS, GPX4, and LPO were assessed. GSH acts as a co‐factor in GPX4‐catalyzed lipid repair systems, and GSH depletion can inactivate GPX4 to boost ferroptosis.^[^ ^9c^ ^]^ As shown in **Figure** **3**A, GSH was depleted significantly (about 50%) by CTFP and CTFAP due to the existence of Fe^3+^, which can convert GSH into GSSG. The Fe^3+^ was translated into Fe^2+^ to enhance the •OH generation by a Fenton‐like reaction.^[^ ^21^ ^]^ GSH is a substance that GPX4 uses to reduce LPO. Therefore, depletion of GSH can decrease GPX4 expression to promote LPO production.^[^ ^22^ ^]^ As a result of the consumption of GSH by Fe^3+^, the expression of GPX4 was reduced with CTFP or CTFAP treatment (Figure 3B; Figure S15A, Supporting Information). Then C11‐BODIPY^581/591^ was introduced as an indicator to detect intracellular LPO levels. The red fluorescence almost disappeared with an intense green fluorescence turn‐up in CTFAP‐treated cells, confirming a significant elevation of LPO generation (Figure S16, Supporting Information). The addition of IFN‐γ further augmented CTFAP‐induced LPO to promote cell ferroptosis through IFN‐γ‐mediated ACSL4 activation. As shown in Figure 3C and Figure S15B (Supporting Information), the expression of ACSL4 increased with IFN‐γ supplementation, facilitating the conversion of AA into AA‐CoA through esterification with coenzyme A (CoA). These derivatives integrate with phospholipids, serving as a precursor for LPO to enhance cell ferroptosis. As an important marker of ferroptosis,^[^ ^14^ ^]^ the increased ACSL4 in CTFAP treated cells further conforming CTFAP triggered ferroptosis in 4T1 cells (Figure 3C). Moreover, the mutual promotion of IFN‐γ and ferroptosis further enhanced expression of ACSL4 in CTFAP and IFN‐γ treated 4T1 cells. Therefore, CTFAP can achieve the mutual promotion of ferroptosis and cancer immunotherapy by AA delivery and IFN‐γ‐mediated ACSL4 regulation.

As a typical sonosensitizer, the existence of TCPP in CTFAP makes it possible for SDT.^[^ ^23^ ^]^ The cell viability of CTFAP has decreased to 17.6% with the application of US (Figure 3D), consistent with the results observed in the live‐dead dual staining results (Figure S17, Supporting Information). The increased cell‐killing ability of CTFAP may be attributed to the enhanced ROS generation by US irradiation. As shown in Figure S18 (Supporting Information), A significant amount of ROS was detected in CTFAP‐treated cells followed by US irradiation. Compared to the CTFAP group, the highest level of LPO was found in the CTFAP + US group (Figure 3E; Figure S16, Supporting Information), they are performed in the same conditions with the same PBS group. These results suggest that the application of the US can promote cell ferroptosis.

### 2.5 In Vitro Evaluation of ICD Triggered by CTFAP with US Irradiation

It has been reported that both SDT and ferroptosis can induce cell ICD.^[^ ^24^ ^]^ The calreticulin (CRT) exposure, adenosine triphosphate (ATP) secretion, and high mobility group protein B1 (HMGB1) release are regarded as the characteristics of ICD occurrence.^[^ ^5a^ ^]^ High expression of CRT on the surfaces of dying tumor cells is one of the most significant signals of ICD, acting as an “eat‐me” signal to stimulate antigen‐presenting cells (APCs) to engulf dying cell fragments.^[^ ^25^ ^]^ As illustrated in Figure 3F, a substantial quantity of CRT (red fluorescence) was observed on the surface of the plasma membrane; quantitative results demonstrated that the CTFAP + US group exhibited approximately eightfold higher CRT exposure than TCPP or CaO_2_ with US irradiation (Figure S19, Supporting Information) due to the enhanced ferroptosis. The released HMGB1 and ATP in the extracellular matrix act as an immune cell attractor to bind APCs and promote immune activation.^[^ ^26^ ^]^ The concentration of HMGB1 and ATP was found to be elevated in the cell medium of the CTFAP + US group (Figure 3G,H). Furthermore, the immunofluorescence intensity of HMGB1 (green fluorescence) was significantly reduced in the CTFAP + US group (Figure S20, Supporting Information), indicating the release of HMGB1 and ATP from tumor cells. These findings indicated that the US‐facilitated ferroptosis can effectively induce cell ICD, which has great potential to promote the maturation of DCs and further stimulate T cells’ activation.

### 2.6 In Vivo Imaging and Anti‐Tumor Efficiency Evaluations

**Figure 4:**
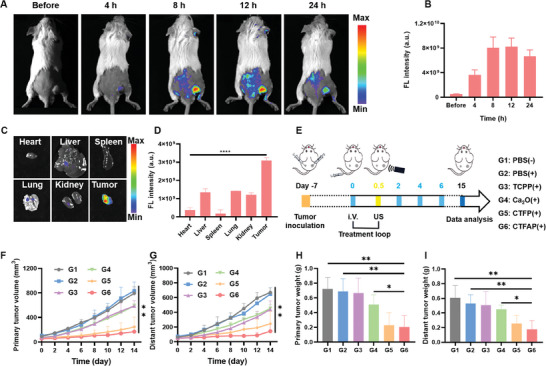
In vivo fluorescence imaging and antitumor activity of CTFAP with US irradiation. A) Fluorescence images and B) corresponding fluorescence intensity of 4T1 tumor‐bearing mice pre and post‐injected with CTFAP. C) Fluorescence images and D) corresponding fluorescence intensity of ex vivo tumors and other organs after 12 h monitoring. E) Schematic illustration of CTFAP and US treated process, “+” represents US irradiation, and “‐” represents without US irradiation. Tumor volume variation curves of the F) primary and G) distant tumors after different treatments. Tumor weight of H) primary and I) distant tumors after different treatments. G1: PBS (‐), G2: PBS (+), G3: TCPP (+), G4: CaO_2_ (+), G5: CTFP (+), G6: CTFAP (+). (mean ± SD, n = 4), *p < 0.05, **p < 0.01, ***p < 0.001, and ****p < 0.0001.

Before anti‐tumor experiments, the tumor accumulation and in vitro biodistribution of CTFAP were monitored in 4T1 tumor‐bearing mice. As shown in **Figure** **4**A, the fluorescence intensity in the tumor region gradually increased over time after intravenous (i.v.) injection of CTFAP (TCPP = 5 mg kg^−1^), reaching a plateau at ≈12 h (Figure 4B). This observed trend serves as effective visual guidance for precise US treatment. Besides, the tumor tissues exhibited the strongest fluorescent signal in comparison to major organs including the liver, heart, spleen, lung, and kidney (Figure 4C,D). The excellent tumor‐targeting capacities of CTFAP may be attributed to the optimized size and good solubility, which are favorable for the enhanced permeation and retention (EPR) effect and reduced clearance in pharmacokinetics.^[^ ^27^ ^]^

Subsequently, the anti‐tumor efficiency of CTFAP was evaluated on the bilateral subcutaneous 4T1 tumor model in female BABL/c mice. The 4T1 cells were implanted bilaterally on the mouse's back (Figure 4E). Tumors on the right side were considered as primary, while those on the left were considered as distant. 7 days after implantation, the tumor‐bearing mice were intravenously injected with PBS, TCPP, CaO_2_, CTFP, and CTFAP followed by US treatment after 12 h post‐administration. The treatment processes were repeated on day 0, 2, 4, and 6. After 15 days, lymph nodes, tumor tissue, and serum were harvested for immunological analysis.

Similar to the PBS group, US irradiation exhibited a negligible therapeutic effect in both primary and distant tumors (Figure 4F,G). Both TCPP and CaO_2_ with US irradiation groups showed an equal degree of tumor suppression, attributed to the SDT effect of TCPP and the elevated H_2_O_2_ by CaO_2_, respectively. As anticipated, both the CTFP and CTFAP exposed to US irradiation groups could significantly delay tumor growth due to the SDT and the Fe^3+^‐mediated ferroptosis. While, the presence of AA (G6) can slightly enhance the therapeutic effect, owing to its promoting effect on LPO. Comparable results could be observed from both primary and distant tumor weight (Figure 4H,I) and photographs (Figure S21, Supporting Information) in each group after 15 days of monitoring. Moreover, no obvious abnormal changes after different treatments were observed in the body weight and pathological analysis of major organs (Figures S22A and S23, Supporting Information), the typical biomarkers for blood biochemistry were all within the normal range (Figure S22B, Supporting Information), and the hemolysis of CTFAP was less than 5% (Figure S24, Supporting Information). The above results indicated that CTFAP possesses excellent antitumor effects with good biocompatibility, which has enormous potential for clinical application.

**Figure 5:**
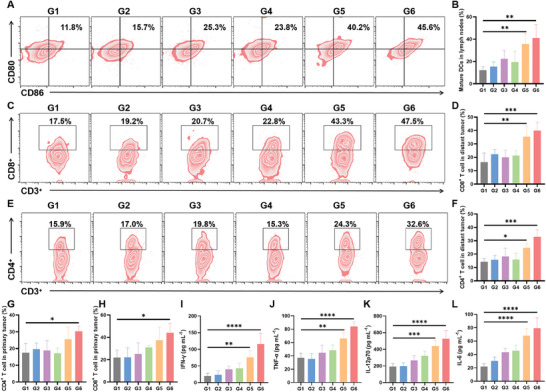
Activation of immune response induced by CTFAP with US irradiation. A) Flow cytometry plots and B) content of matured DCs (CD11c^+^CD80^+^ CD86^+^) in lymph nodes with varying treatments. C) Flow cytometry plots and D) corresponding CD8^+^ T cells (CD45^+^CD3^+^CD8^+^) content in distant tumors with varying treatments. E) Flow cytometry analysis and F) relative content of CD4^+^ T cells (CD45^+^CD3^+^CD4^+^) in distant tumors with varying treatments. Quantitative analysis of G) CD4^+^ and H) CD8^+^ T cells in primary tumors. I‐L) IFN‐γ, TNF‐α, IL‐12p70, and IL‐6 levels in serum of treated mice. G1: PBS (‐), G2: PBS (+), G3: TCPP (+), G4: CaO_2_ (+), G5: CTFP (+), G6: CTFAP (+). “+” represents US irradiation, “‐” represents without US irradiation. (mean ± SD, n = 4), *p < 0.05, **p < 0.01, ***p < 0.001, and ****p < 0.0001.

The good therapeutic effect on distant tumors may be attributed to the activation of the immune response. Therefore, we evaluated the immunological effect of CTFAP under US irradiation. The mature DCs (CD80^+^ CD86^+^cells gated on CD11c^+^ lymphocytes) percentage in the CTFAP with US irradiation group (G6) was about twice that of the PBS group (G1) due to the enhanced cancer‐immunogenic ferroptosis mediated by AA (**Figure** **5**A,B; Figure S25, Supporting Information). The mature DCs could promote T‐cell activation for anti‐tumor immune response.^[^ ^28^ ^]^ The levels of helper T cells (CD4^+^ T cells) and cytotoxic T cells (CD8^+^ T cells) in tumors were further measured through flow cytometry. As shown in Figure 5C–H and Figure S26 (Supporting Information), no significant differences were observed in PBS, PBS (+), TCPP (+), and CaO_2_ (+) groups, while the introduction of Fe^3+^ (G5) led to an increase in the levels of CD4^+^ T (≈25.5%) and CD8^+^ T (≈37.4%). With the AA‐mediated ferroptosis and immune mutually promoting (G6), CD4^+^ T and CD8^+^ T levels were further increased to ≈30.2% and ≈44.0%, respectively (Figure 5G,H; Figure S26 and S27, Supporting Information). Comparable results could be observed in Figure 5C–F, the CTFAP with US irradiation group (G6) could effectively activate T cells for tumor killing. Moreover, as the key factors for tumor inhibition, the pro‐inflammatory cytokines (IFN‐γ, TNF‐α, IL‐12p70, and IL‐6) in the serum of treated mice were evaluated by ELISA kits. Compared to the PBS group, the CTFAP exposed to the US irradiation group (G6) significantly increased the levels of the pro‐inflammatory cytokine in sera (Figure 5I–L), demonstrating that the Fe^3+^ and CaO_2_‐based cascade reaction and AA‐mediated ferroptosis enhancement can boost the systemic immune response.

## 3 Conclusion

In summary, we have developed an integrated nanosystem (CTFAP) capable of robust ROS generation coupled with ferroptotic lipid modulation to achieve mutual enhancement of ferroptosis and immunotherapy. This CaO_2_ core‐based nanosystem can provide O_2_ for TCPP‐based SDT and generate H_2_O_2_ for Fe^3+^‐mediated Fenton‐like reaction, leading to robust ROS generation for ferroptosis enhancement. In addition, this process induces cell ICD, promoting DC maturation and stimulating systemic antitumor immune responses, including IFN‐γ secretion. The released IFN‐γ not only inhibits the expression of GPX4 but also increases the expression of ACSL4. The liberation of AA from CTFAP nanoparticles further accelerated the accumulation of LPO, thereby inducing ferroptosis in cancer cells. In vivo results demonstrated that CTFAP nanoparticles effectively suppressed the growth of primary and distant tumors with favorable biosafety profiles. Therefore, the strategy of mutually enhancing ferroptosis and antitumor immunity offers promising perspectives for the development of immunomodulatory anticancer candidates.

## Conflict of Interest

The authors declare no conflict of interest.

## Supporting information



Supporting Information

## Data Availability

The data that support the findings of this study are available from the corresponding author upon reasonable request.
