# Supplementary material for: A Robust ROS Generation and Ferroptotic Lipid Modulation Nanosystem for Mutual Reinforcement of Ferroptosis and Cancer Immunotherapy
Source: Adv Healthc Mater. 2024 Oct 1;13(30):2401502. doi: 10.1002/adhm.202401502 (PMC11616257; doi:10.1002/adhm.202401502)
Supplement: Supplementary file 1 — Supporting Information [file ADHM-13-0-s001.docx]

Supporting information

A robust ROS generation and ferroptotic lipid modulation nanosystem for mutual reinforcement of ferroptosis and cancer immunotherapy

*Chao Jiang, Wenxi Li, Jie Yan, Xinying Yu, Yuzhao Feng, Bei Li, Yuan Liu,* Yunlu Dai**

C. Jiang, W. Li, Jie Yan, X. Yu, Y. Feng, B. Li, Prof. Y. Liu, Prof. Y. Dai

Cancer Center and Institute of Translational Medicine, Faculty of Health Sciences, University of Macau, Macau 999078, China

E-mail: [yldai@um.edu.mo](mailto:yldai@um.edu.mo)

C. Jiang, W. Li, Jie Yan, X. Yu, Y. Feng, B. Li, Prof. Y. Liu, Prof. Y. Dai

MoE Frontiers Science Center for Precision Oncology, University of Macau, Macau 999078, China

C. Jiang, Prof. Y. Liu

Zhejiang Cancer Hospital, Hangzhou Institute of Medicine (HIM), Chinese Academy of Sciences, Hangzhou 310022, Zhejiang, China

E-mail: [yuanliu@ucas.ac.cn](mailto:yuanliu@ucas.ac.cn)

Materials

Calcium chloride (CaCl_2_) and polyvinyl pyrrolidone (PVP) were acquired from Sigma-Aldrich. mPEG-DSPE (sodium salt) M.W.2000 was procured from Tanshui Technology Co., Ltd. Recombinant murine IFN-γ, ethanol, and Cell Counting Kit-8 (CCK-8) were purchased from MedChemExpress. Arachidonic acid (AA), 1,3-diphenylisobenzofuran (DPBF), 3,3',5,5'-Tetramethylbenzidine (TMB) were purchased from Aladdin Reagent Co. Ltd. (Shanghai, China). Hydrogen peroxide (H_2_O_2_) and aqueous ammonia (NH_3_·H_2_O) were obtained from XiLONG SCIENTIFIC. 2’,7’-Dichlorofluorescein diacetate (DCFH-DA) and BODIPY^581/591^ C11 were purchased from Invitrogen.The Hydrogen Peroxide Assay Kit, Calcein-AM/PI Double Stain Kit, GSH and GSSG Assay Kit, HRP-labeled goat anti-rabbit IgG (H+L), and ECL western blotting system were purchased from Beyotime. GAPDH (ab8245), ACSL4 (ab155282), and GPX4 (ab125066) antibodies were obtained from Abcam. All reagents were used without further purification.

**Preparation of CaO_2_@TCPP/Fe-AA@PEG (CTFAP)**

pH-responsive CaO_2_ nanoparticles were first prepared following a reported method.^[1]^ Then, AA (25 μL, 40 mg/mL) and TCPP (100 μL, 10 mg/mL) were mixed with 4 mL of the as-prepared CaO_2_ (0.5 mg/mL) under stirring for 5 min, CaO_2_@TCPP/Fe-AA (CTFA) was obtained with the addition of FeCl_3_ (200 μL, 10 mg/mL) under vigorous stirring for 30 min. The obtained CTFA was purified by centrifugation (10000 rpm, 10 min), washed with ethanol twice, and re-dispersed in ethanol. mPEG-DSPE was further introduced to enhance the stability and aqueous solubility of CTFA. Briefly, CTFA was mixed with mPEG-DSPE at the ratio of 1:10 (m/m) and stirred overnight to obtain CaO_2_@TCPP/Fe-AA@PEG (CTFAP). CaO_2_@TCPP/Fe@PEG (CTFP) nanoparticles were prepared following the same steps without the addition of AA. All [reagent](javascript:;)s were dissolved in ethanol.

Characterization of CTFAP

UV-vis-NIR absorption spectra were measured by using ultraviolet-visible (UV-vis) spectroscopy (SHIMADZU UV-1800). Transmission electron microscopy (TEM) images were taken by HT7700 TEM (Hitachi Electronics, Japan). X-ray photoelectron spectroscopy (XPS) was conducted on a Thermo ScientificTM K-AlphaTM+ spectrometer equipped with a monochromatic Al Kα X-ray source (1486.6 eV) operating at 100 W. Zeta potential was measured with a Zetasizer Nano-ZS (Malvern Instruments, UK). Calcium concentration was quantified by an Inductively Coupled Plasma Mass Spectrometry (ICP-MS) (7800, Agilent, USA). Cellular fluorescence was taken by Laser Confocal Microscope SpinSR10 machine (Olympus, Japan).

**In vitro degradation of CTFAP**

A dialysis bag (MWCO = 3500) containing CTFAP (1 mL, 3 mg/mL) was sealed and placed in bakers containing 49 mL of phosphate-buffered saline (PBS) with varying pH (7.4, 6.5, and 5.5). The degradation experiments were then conducted at room temperature under continuous stirring. The amount of Ca^2+^ ions released at different time points (0.5, 1, 2, 4, 8, 12, 24 h) was measured by ICP-MS.

**In vitro hydroxyl radicals (**•OH**) generation**

To investigate the cascade reaction resulting •OH generated by degraded CTFAP. 30 μg/mL of CTFAP was added into a PBS buffer containing 0.8 mM TMB at different pH (7.4, 6.5, and 5.5) for 1 h. For concentration-dependent •OH generation, different concentrations of CTFAP were incubated with 0.8 mM TMB at pH 6.5. The UV changes of the solution were recorded by UV-vis.

**In vitro cell experiments**

In vitro cell cytotoxicity experiments were conducted on 4T1 cells. The cells were seeded in 96-well plates at a density of 5×10^3^ cells per well and incubated at 37 ºC in RPMI 1640 medium containing 10% fetal bovine serum (FBS) under a 5% CO2 atmosphere for 24 h. Following incubation, the cells were washed with PBS and co-incubated with varying concentrations of CaO_2_, TCPP, CTFP, and CTFAP in PBS for an additional 24 h under the same conditions. After the incubation, the relative cell viability was assessed by using the CCK-8 assay. For pH-responsive cell toxicity, the cells were exposed to different concentrations of CTFAP (20 μg/mL) in RPMI 1640 with varying pH levels (7.4 and 6.5) for 24 h. For in vitro ultrasound (US)-enhanced therapy, the cells were treated with CaO_2_, TCPP, CTFP, and CTFAP (20 μg/mL) for 6 hours, followed by exposure to US irradiation (1.0 MHz, 1.5 W/cm^2^, 50% duty cycle) for 3 min. Afterward, the cells were cultured for another 18 h. Subsequently, the CCK-8 assay was used to measure the relative cell viability. For the dual staining of live and dead cells, the cells subjected to treatment were exposed to staining reagents, specifically CA (4 μM) and PI (4 μM), for 5 min. To determine the production of ROS, the treated cells were exposed to RPMI 1640 without FBS containing DCFH-DA (10 μM) for 30 min. For the detection of intracellular lipid peroxidation (LPO), the treated cells were incubated with C11-BODIPY^581/591^ (10 μM) for 30 min. All the fluorescence images were captured by using a confocal laser scanning microscopy (SpinSR10, Olympus).

**Intracellular H_2_O_2_ generation and GSH depletion detection**

4T1 cells were seeded in 6-well plates at a density of 1×10^5^ cells per well and incubated at 37 ºC in RPMI 1640 medium with 10% FBS under a 5% CO_2_ atmosphere for 24 h. Subsequently, cells were washed with PBS and exposed to PBS, CaO_2_, TCPP, CTFP, and CTFAP for 8 h. The cells were collected and lysed using cell lysis buffer. The supernatant was collected by centrifugation and analyzed using a Hydrogen Peroxide Assay Kit and a GSSG Assay Kit, respectively.

**Immunogenic cell death caused by CTFAP**

4T1 cells were seeded in 12-well plates at a density of 3×10^4^ cells per well for 24 h. After washing with PBS, cells were treated with PBS, CaO_2_, TCPP, CTFP, and CTFAP for 6 h and exposed to US irradiation (1.0 MHz, 1.5 W/cm^2^, 50% duty cycle, 3 min). After a further 18 h incubation, the supernatant was collected for the detection of released ATP. The treated cells were stained by Alexa Fluor® 647 labeled anti-CRT antibody (eBioscience) or Alexan Fluor 555 labeled HMGB1 antibody (eBioscience). Subsequently, the cells were washed with PBS three times, fixed with 4% PFA, stained with DAPI, and finally observed under confocal laser scanning microscopy (SpinSR10, Olympus).

Western blot analysis

The 4T1 cells were treated with PBS, CaO_2_, TCPP, CTFP, and CTFAP before being lysed by RIPA lysis buffer containing 1% phenylmethanesulfonylfluoride (PMSF). The resulting mixture was centrifuged to collect the supernatant (12000 rpm for 10 min at 4°C). The protein content was measured by using the BCA assay kit and loaded onto an SDS-PAGE gel for electrophoresis. The gel was then transferred to a polyvinylidene fluoride (PVDF) membrane. After blocking the PVDF membranes with 5% bovine serum albumin (BSA) for 1 h, followed by washing three times with tris-buffered saline containing 0.1% Tween 20 buffer (TBST). Then they were co incubated with specific primary antibodies overnight at 4°C followed by washing three times with TBST. After incubation with goat anti-rabbit HRP secondary antibodies took place at room temperature for 1 h. The signal of each marker was detected by using BeyoECL Moon and observed through Amersham ImageQuant 800 (Cytiva, USA).

**Animal model**

All experiments involving animals were authorized by the University of Macau Animal Ethics Committee (UMARE-030-2018). Female BABL/c mice aged 4-5 weeks were implanted subcutaneously with 1 x 10^6 and 0.5 x 10^6 of 4T1 cells. The largest length and smallest width of the tumors were assessed using digital vernier calipers. Tumor volumes were calculated by applying the following formula: Volume = (tumor length) × (tumor width) ^2 / 2.

**In vivo biodistribution**

The biodistribution of CTFAP was analyzed by using a unilateral tumor model. When the tumor size reached approximately 80 mm^3^, CTFAP (5 mg/kg of TCPP) was administered to the 4T1 tumor-bearing mice intravenously. [Fluorescence](javascript:;) signals of tumor tissues were monitored at different time points (0, 4, 8, 12, and 24 h post-injection) by utilizing an IVIS Lumina XR III imaging system (Caliper Life Sciences).

**In vivo treatment.**

7 days after tumor cells implantation, the tumor-bearing mice were randomly assigned into six groups (n = 4): G1: PBS, G2: PBS + US, G3: TCPP + US, G4: CaO_2_ + US, G5: CTFP + US and G6: CTFAP + US (5 mg/kg of TCPP and 9.2 mg/kg of CaO_2_). The tumors were exposed to a US irradiation (1.0 MHz, 2.0 W/cm^2^, 50% duty cycle) for 5 min at post-inject 12 h. The treatment protocol was administered four times, on days 0, 2, 4, and 6, respectively. Tumor volume and mice body weight were recorded bi-daily throughout. The mice were euthanized on day 15, and the tumor tissue, lymph nodes, and serum were collected for immunological and biochemical analysis.

**Analysis of anti-tumor activity**

The lymph nodes and tumor samples collected from mice were processed using a previously described method to create a single-cell suspension.^[2]^ Afterward, dye-labeled antibodies were used to stain the single cells for flow cytometry analysis. To assess DCs maturation in the lymph nodes, the single cells of lymph nodes were stained with FITC anti-mouse CD11c (eBioscience), PE anti-mouse CD80 antibodies (eBioscience), and APC anti-mouse CD86 (eBioscience). For the content of CD8^+^ and CD4^+^ T cells detection, single cells from tumors were stained by FITC anti-mouse CD45 (eBioscience), PE anti-mouse CD3e (eBioscience), APC anti-mouse CD4 (eBioscience)and AF700 anti-mouse CD8a (eBioscience). The collected serum samples were diluted to detect the levels of excreted cytokines by using an ELISA Kit (NeoBioscience).

**Hemolysis Assay**

The different concentrations of CTFAP (0, 12.5, 25, 50, 100, 200, 400 μg/mL or water) were incubated with purified red cells for 3 h. The hemolysis ratio was calculated based on the absorbance of the upper supernatants at 541 nm using the following equation:

Hemolysis = (A _sample_ - A_-_) / (A_+_ - A_-_) *100%

**Statistical analysis**

All data were presented as mean ± standard deviation (SD). Statistical analysis was conducted by using a one-way ANOVA, followed by Tukey’s post hoc test, with GraphPad Prism 8.0. Statistical significance was considered for P values below 0.05 (*p < 0.05, **p < 0.01, ***p< 0.001, and ****p< 0.0001).

**References:**

[1] S. Shen, M. Mamat, S. C. Zhang, J. Cao, Z. D. Hood, L. Figueroa-Cosme, Y. N. Xia, *Small* **2019**, 15, 1902118.

[2] J. Yan, W. X. Li, H. Tian, B. Li, X. Y. Yu, G. H. Wang, W. Sang, Y. L. Dai, *ACS Nano* **2023**, 17, 14667.

**Results and Data:**


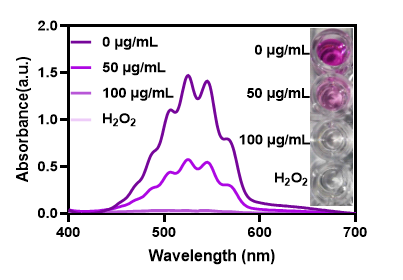


**Figure S1.** Color and UV-vis spectra change of potassium permanganate (KMnO_4_) (50 μg/mL) solution containing H_2_SO_4_ (0.1 M) incubated with various concentrations of CaO_2_ nanoparticles with or H_2_O_2_ (1 wt %) for 5 min.


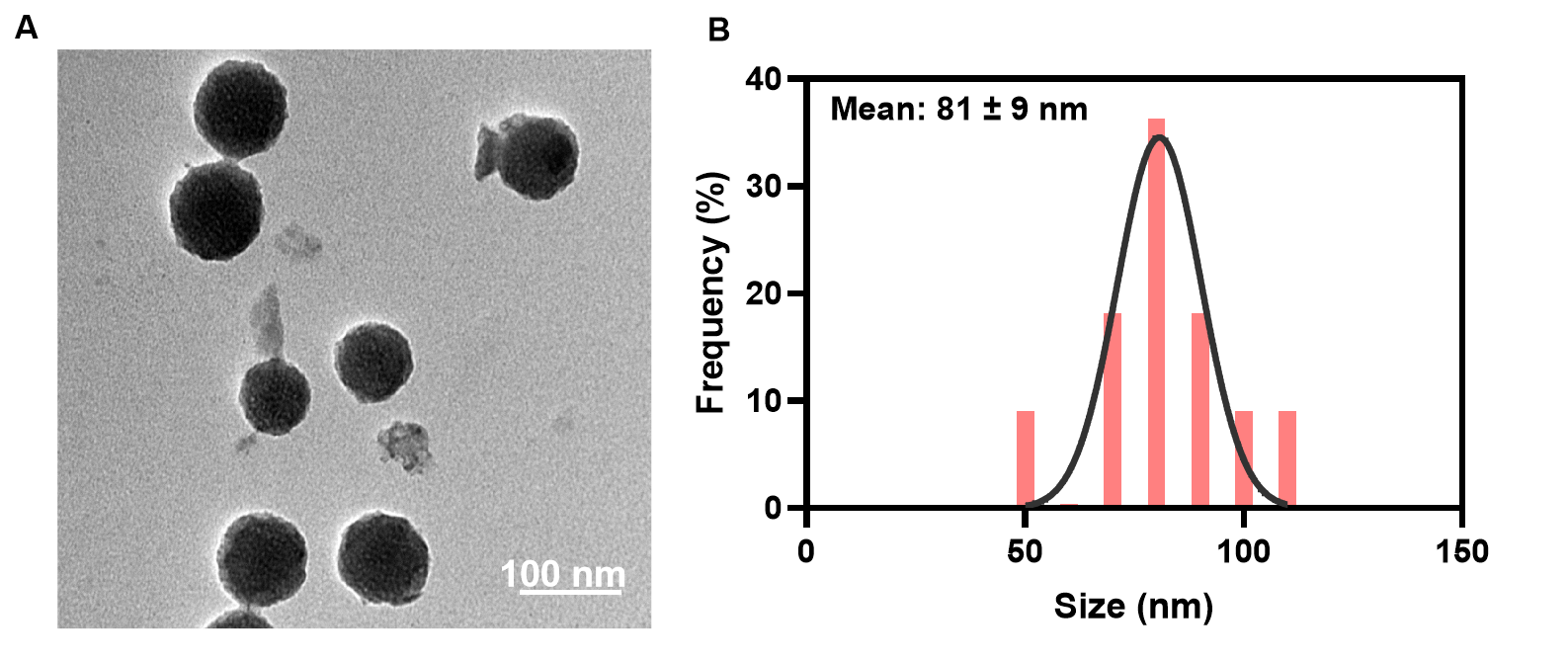


**Figure S2. (A)** TEM images of CaO_2_ and (B) corresponding diameter distribution. Scale bar: 100 nm.


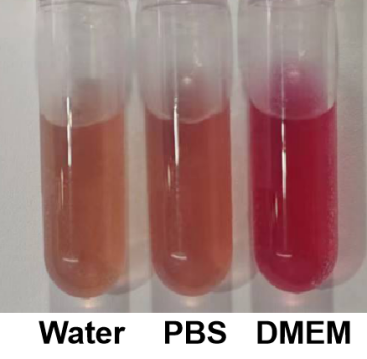


**Figure S3.** The Photographs of CTFAP in different physiological media.


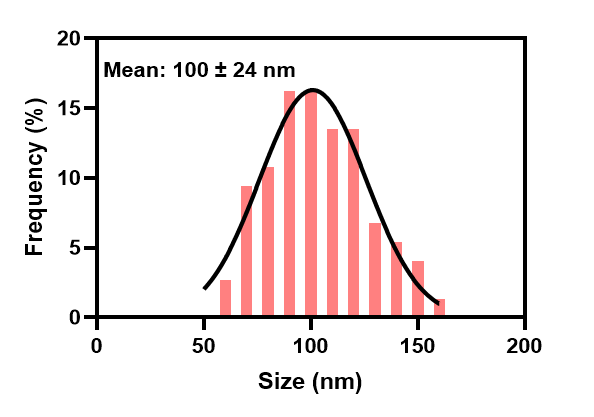


**Figure S4.** The diameter distribution of CTFAP obtained from Figure 1a.


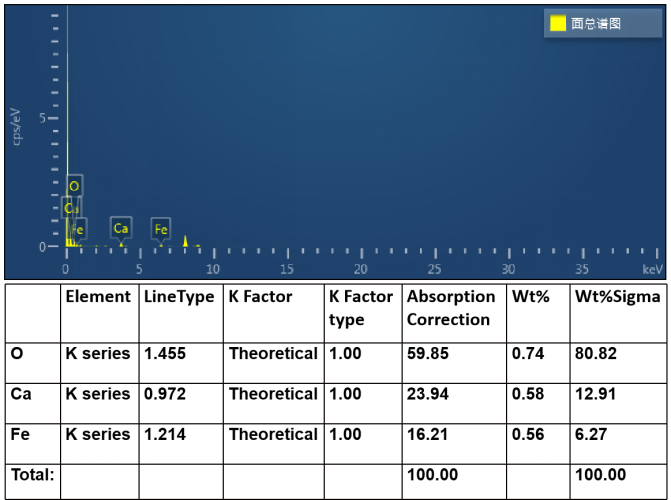


**Figure S5.** EDS spectrum of CTFAP and analysis data.


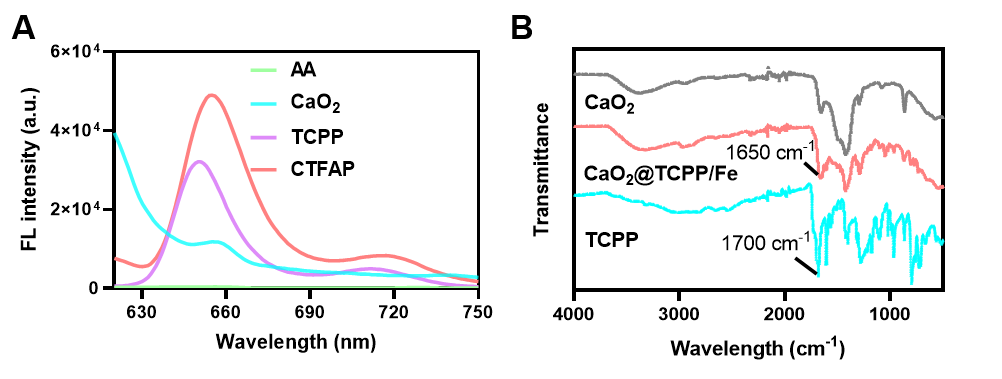


**Figure S6.** (A) [Fluorescence](javascript:;) spectra of AA, CaO_2_, TCPP, and CTFAP. (B) FTIR spectrum of CaO_2_, CaO_2_@TCPP/Fe, and TCPP.


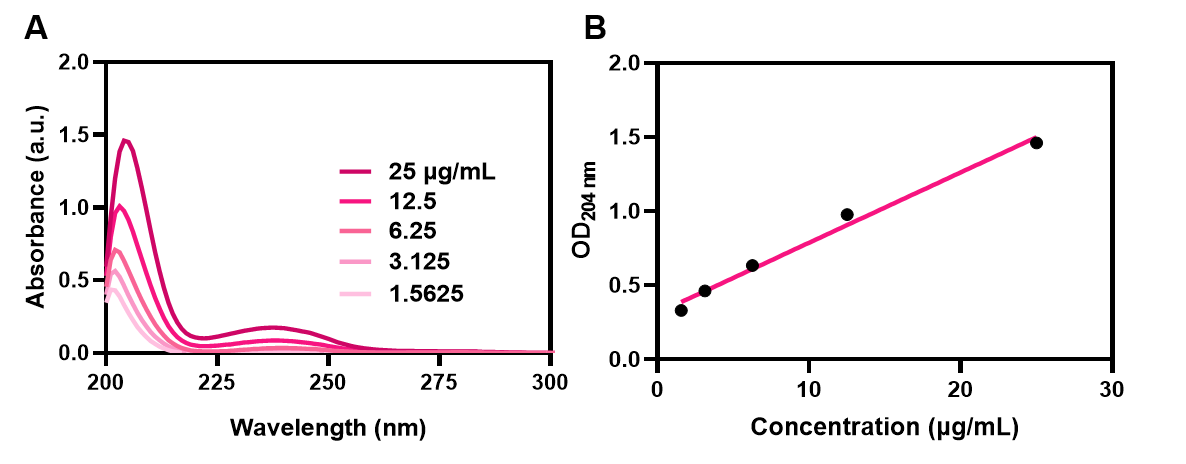


**Figure S7.** (A) UV-vis spectra of different concentrations of AA. (B) Linear relationship of OD_204_ nm with different concentrations of AA.


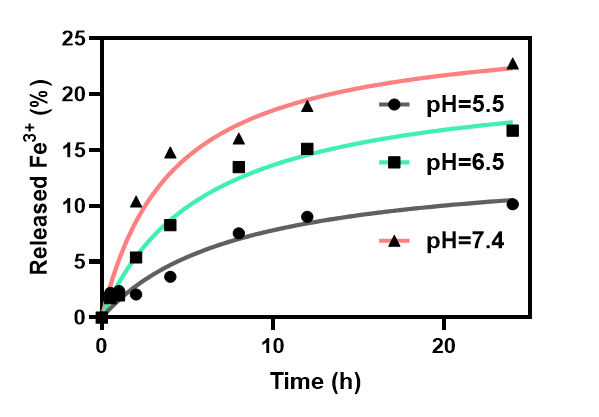


**Figure S8.** Fe^3+^ release profiles of CTFAP in phosphate-buffered saline with varying pH.


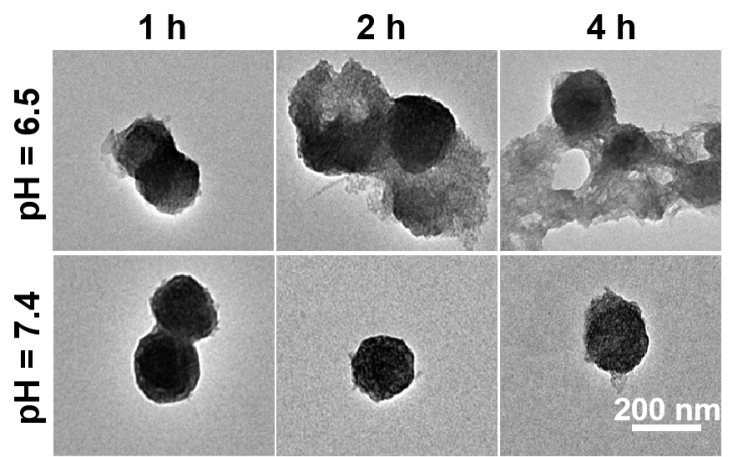


**Figure S9.** The TEM images of CTFAP incubated with PBS with different pH. Scale bar: 200 nm.


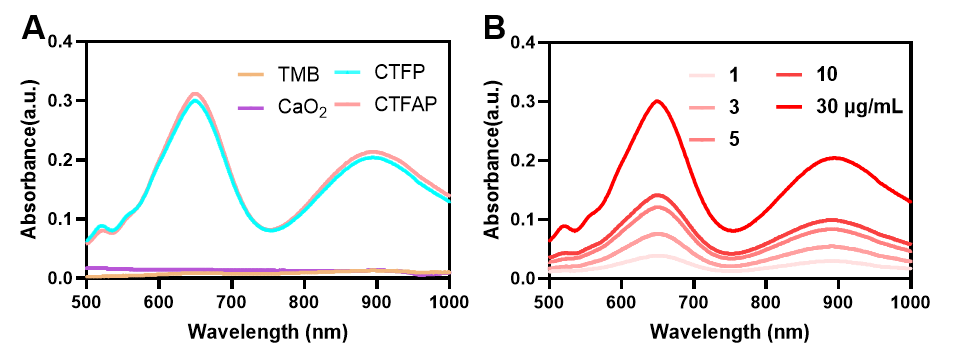


**Figure S10.** (A) UV-vis spectra of TMB, CaO_2_ + TMB, CTFP + TMB, and CTFAP + TMB at acid condition (pH=6.5). (B) UV-vis spectra of CTFAP + TMB (CTFAP concentration: 1, 3, 5, 10, and 30 µg/mL) at acid condition (pH=6.5).


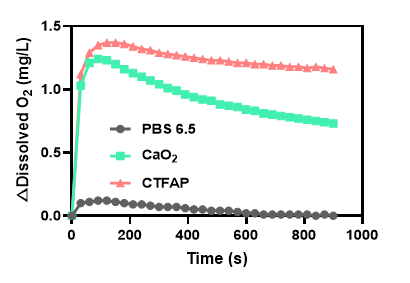


**Figure S11.** O_2_ concentration measurement of CaO_2_ and CTFAP in PBS at pH 6.5.


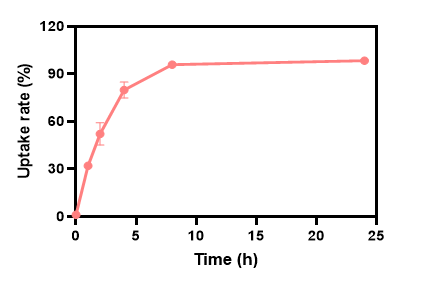


**Figure S12.** Quantitative results of the cell uptake rate of CTFAP incubated with 4T1 cells for 0, 1, 2, 4, 8, and 24 h.


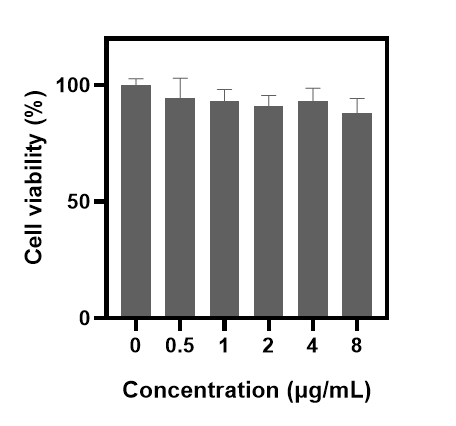


**Figure S13.** Viabilities of 4T1 cells with various amounts of AA.


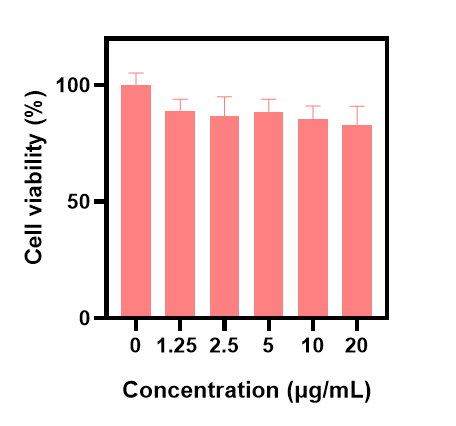


**Figure S14.** Viabilities of MCF-10A cells with various amounts of CTFAP.


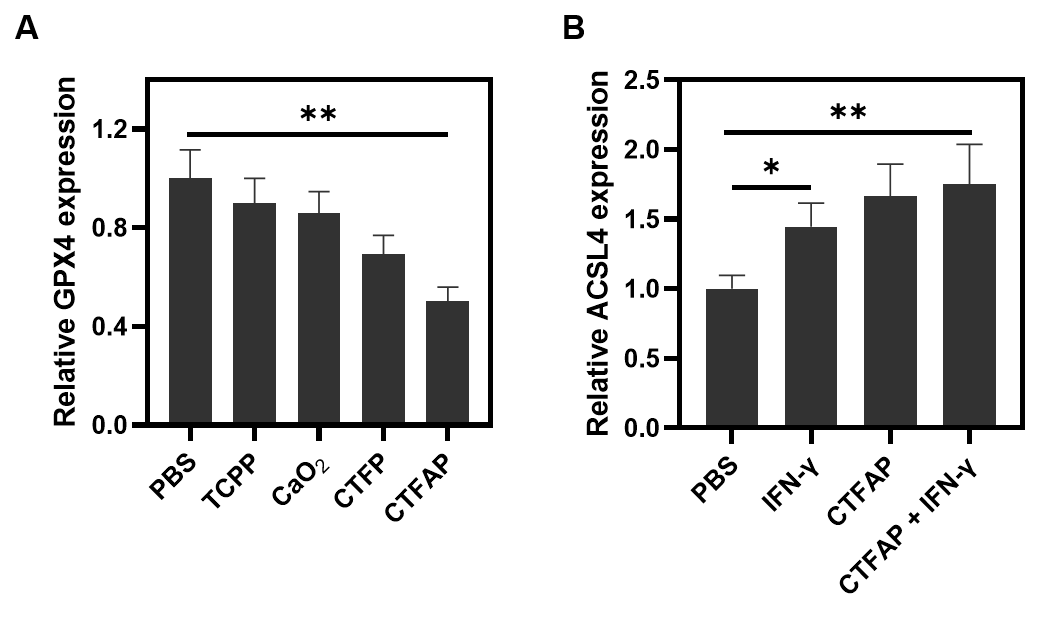


**Figure S15.** The relative expression of (A) GPX4 and (B) ACSL4 after various treatments.


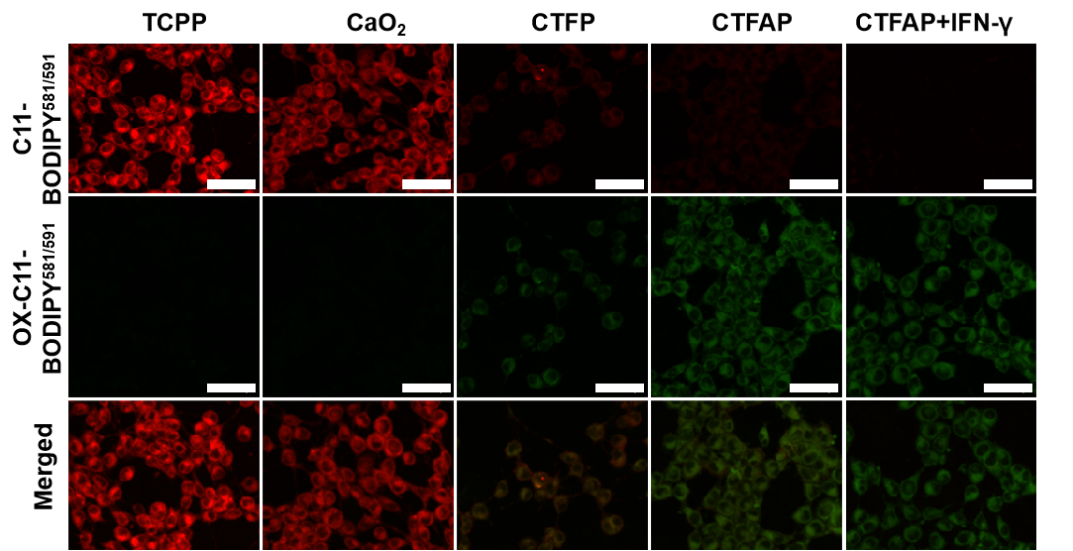


**Figure S16.** Detection of intracellular LPO by C11-BODIPY^581/591^ probe after incubation with PBS, TCPP, CaO_2_, CTFP, CATFP, and CATFP plus IFN-γ. Scale bar: 50 μm.


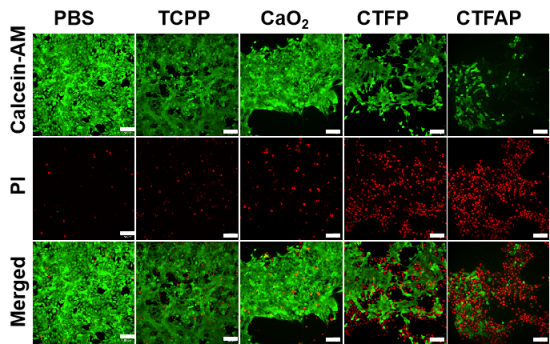


**Figure S17.** Microscopy images of 4T1 cells co-stained with calcein-AM (live cells: green) and PI (dead cell: red) after incubation with PBS, TCPP, CaO_2_, CTFP, and CTFAP with US irradiation. Scale bar: 100 μm.


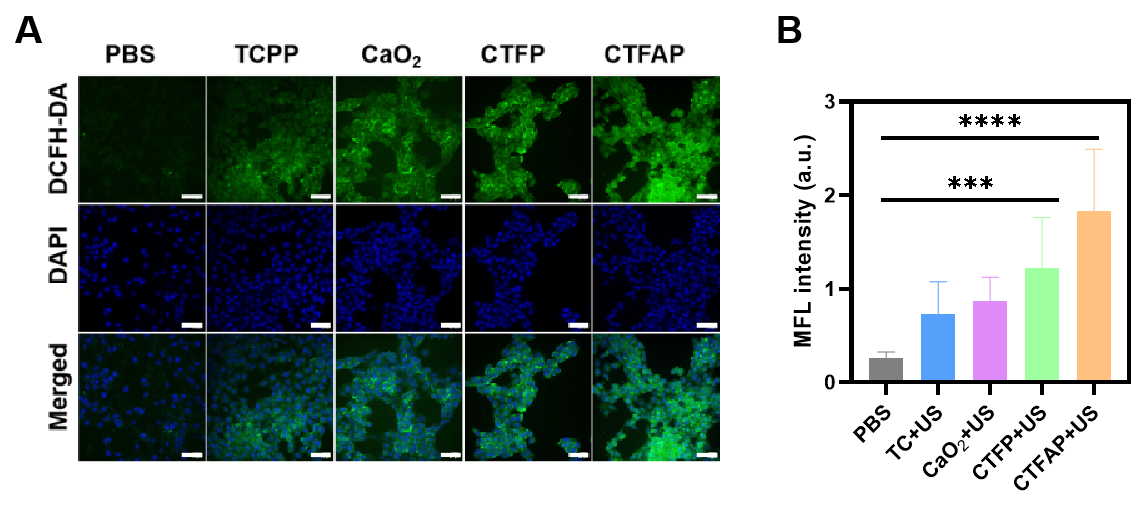


**Figure S18.** (A) Microscopy images and (B) corresponding mean fluorescence intensity of DCFH-DA in 4T1 cells after incubation with PBS, TCPP, CaO_2_, CTFP, and CTFAP with US irradiation. Scale bar: 50 μm.


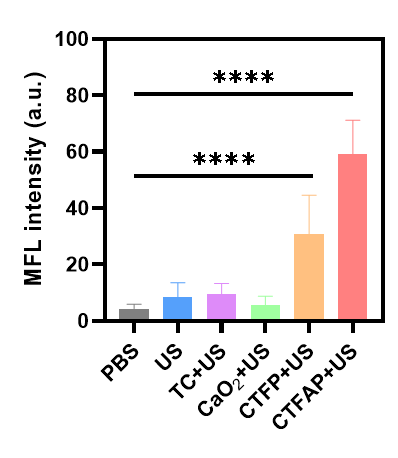


**Figure S19.** Quantification of fluorescence intensities in Figure 3B.


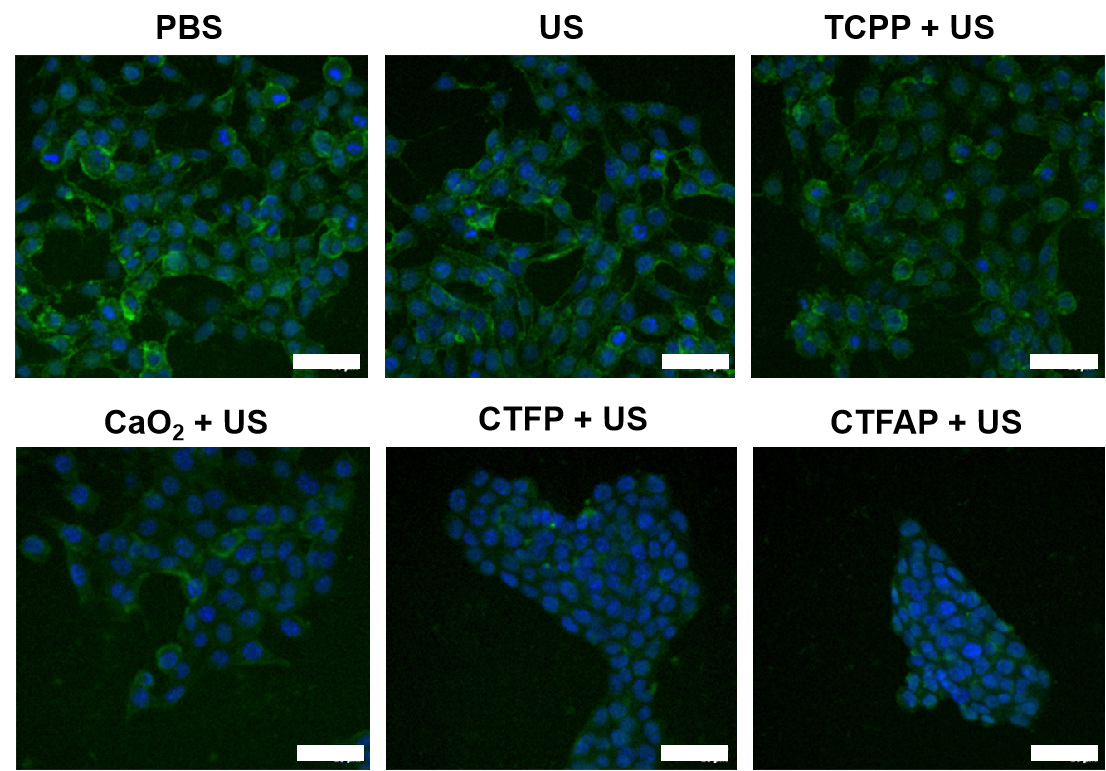


**Figure S20.** Microscopy images of HMGB1 (green) in 4T1 cells after various treatments. Scale bar: 50 μm.


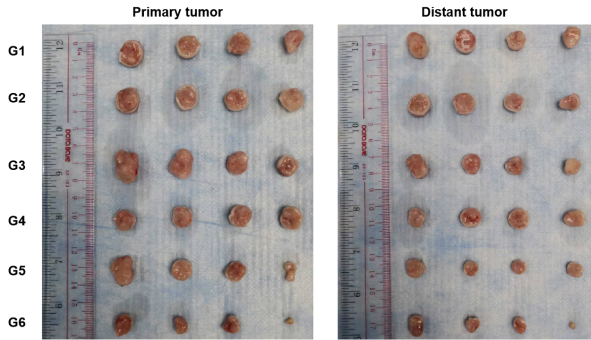


**Figure S21.** Photographs of ex vivo primary and distant tumors after different treatments. G1: PBS (-), G2: PBS (+), G3: TCPP (+), G4: CaO_2_ (+), G5: CTFP (+), G6: CTFAP (+). ‘+’ stands for US irradiation, and ‘-’ represents without US irradiation (1.0 MHz, 2.0 W/cm, 50% duty cycle) for 5 min.


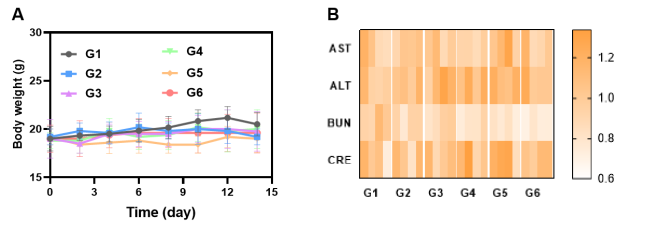


**Figure S22.** (A) The mice body weight curve of each group during the treatment. (B) The corresponding heatmap of AST, ALT, BUN, and CRE levels in serum after different treatments. The color variance represents the biochemical analysis data.


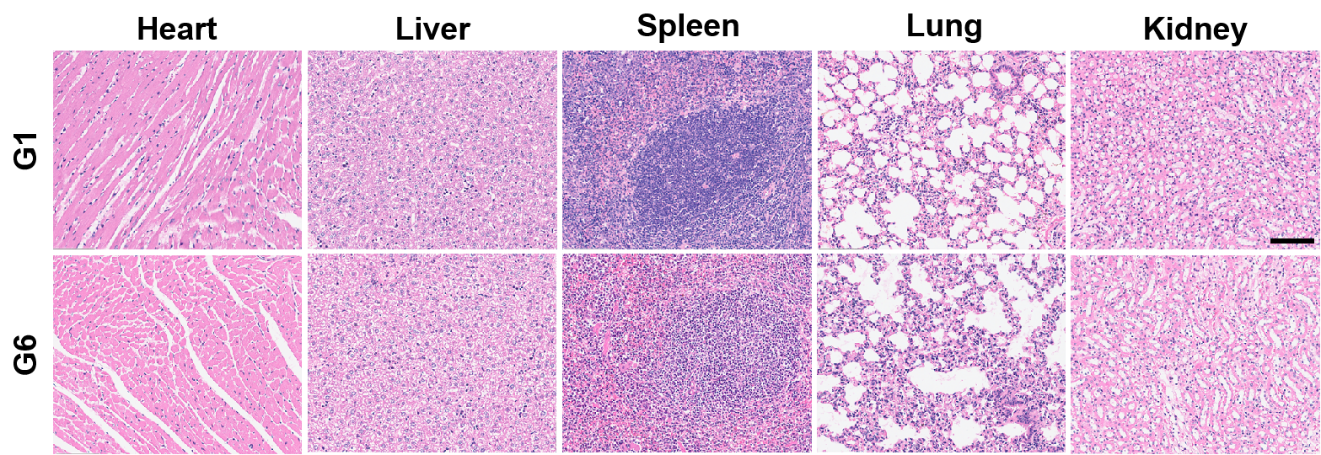


**Figure S23.** Hematoxylin and eosin (H&E) staining of main organs from G1:PBS and G6 CTFAP + US. Scale bar: 100 µm


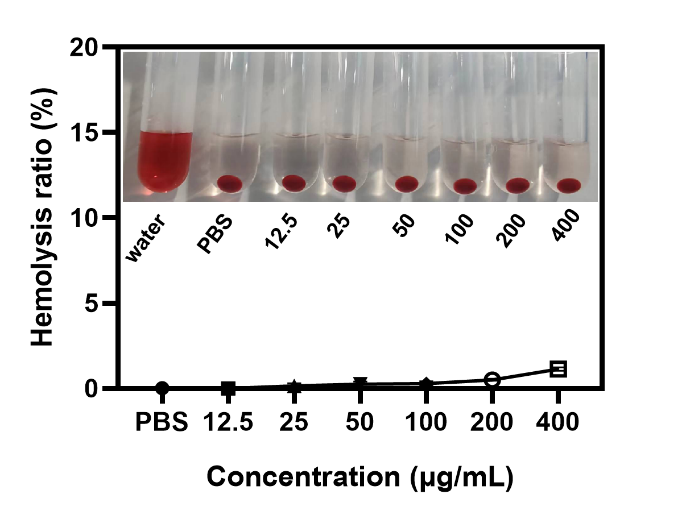


**Figure S24.** The hemolysis of CTFAP.


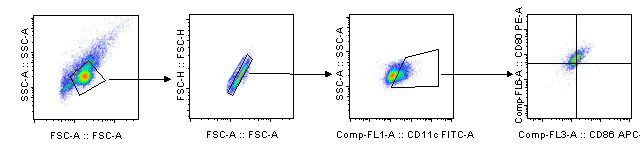


**Figure S25.** Representative flow cytometry gating manner for mature DCs (CD11c^+^CD80^+^CD86^+^).


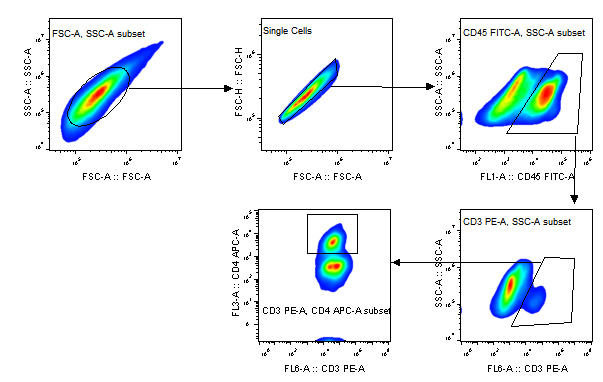


**Figure S26.** Representative flow cytometry gating manner for CD4^+^ T cells (CD45^+^CD3e^+^CD4^+^), CD8^+^ T cells (CD45^+^CD3e^+^CD8a^+^).


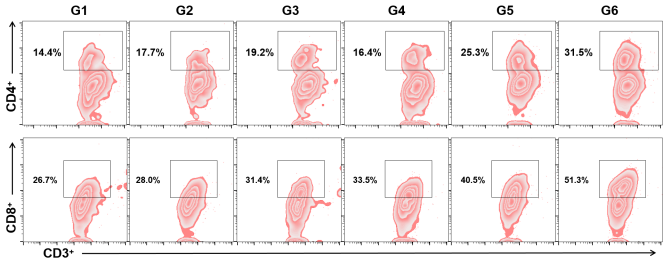


**Figure S27.** Flow cytometric assay and relative populations of CD4^+^ and CD8^+^ T cells in primary tumor after different treatments.
